# IL-6 influences the polarization of macrophages and the formation and growth of colorectal tumor

**DOI:** 10.18632/oncotarget.24734

**Published:** 2018-04-03

**Authors:** Lechuang Chen, Shuren Wang, Yu Wang, Weina Zhang, Kai Ma, Chenfei Hu, Hongxia Zhu, Shufang Liang, Mei Liu, Ningzhi Xu

**Affiliations:** ^1^ Laboratory of Cell and Molecular Biology and State Key Laboratory of Molecular Oncology, National Cancer Center/Cancer Hospital, Chinese Academy of Medical Sciences and Peking Union Medical College, Beijing, China; ^2^ State Key Laboratory of Biotherapy and Cancer Center, West China Hospital, Sichuan University, and Collaborative Innovation Center for Biotherapy, Chengdu, China

**Keywords:** macrophages, polarization, colon cancer, IL-6, animal model

## Abstract

Macrophages play a crucial role in tumorigenesis depending upon the phenotype of macrophages found in tumor microenvironments. To date, how the tumor microenvironment affects the phenotypes of macrophages is not yet fully understood. In this study, we constructed a NIH3T3/Src cell line stably overexpresses the Src protein and found that conditioned medium from this cell line was able to induce polarization towards the M2 phenotype in primary bone marrow-derived macrophages (BMDM) and Ana-1 macrophages. Further investigation revealed that IL-6 produced by NIH3T3/Src cells plays a key role in M2 polarization. During the development of colorectal cancer in C57BL/6J-Apc^Min/+^ mice, increased IL-6 secretion in the interstitial fluid of the colorectal tissues was observed. Furthermore, tumorigenesis in IL-6^tm1Kopf^ mice treated with AOM-DSS, an IL-6 knockout mouse strain, was significantly inhibited compared with the control group, suggesting the important role of IL-6 in promoting tumorigenicity. Our findings identify the target molecules and proinflammatory cytokines responsible for promoting polarization towards the M2 phenotype in macrophages present in tumor microenvironment, which may be useful for the design of novel therapeutic strategies for colorectal cancer.

## INTRODUCTION

Solid tumors exist in an extremely complicated microenvironment. “Tumor microenvironment” refers to the steady-state local environment during tumor growth, which consists of tumor cells and a variety of other cell types including macrophages [1, 2]. It is well-established that M1 macrophages are involved in the inflammatory response and antitumor immunity whereas M2 macrophages exert anti-inflammatory and pro-tumorigenic activities. Studies have demonstrated that macrophages play an important role in tumorigenesis because of their dual functions in inhibition and promotion of tumor growth that varies with their phenotype [3]. Interestingly, upon stimulation by certain cytokines and secretory growth factors in the tumor microenvironment, macrophages can be polarized to the M2 phenotype, which promotes tumor growth. However, how macrophages are polarized with the tumor microenvironment remains to be fully understood.

Cytokines play a critical role in the polarization of macrophages. Stimulation with IFN-γ secreted by Th1 cells and bacterial lipopolysaccharide (LPS) is needed for the classical activation of macrophages, which produces the M1 phenotype. M1 macrophages express and secrete various pro-inflammatory cytokines, resulting in the effective elimination of tumor cells and invading pathogens. Alternatively, IL-4 and IL-13 produced by Th2 cells induce macrophage polarization towards the M2 phenotype; M2 macrophages secretes anti-inflammatory cytokines such as IL-10, immunosuppressive factors, and tumor growth factors, which promote tumor cell growth and metastasis. M1 and M2 macrophages have a high degree of heterogeneity and plasticity as mutual transformation can occur depending on the environment [4]. Macrophages can also exist in various intermediate states of between M1 or M2-like phenotypes as the environment changes [5, 6].

The NF-κB and JAK/STAT3 pathways are very important in driving cancer-associated inflammation. In an inflammatory environment, the NF-κB pathway is sustainably activated in cancer cells [7], leading to the production of inflammatory factors that can activate and recruit macrophages [8]. STAT3 is believed to be associated with tumor growth because it is steadily activated in tumor cells [9]. Several lines of evidence indicate that the NF-κB and JAK/STAT3 pathways mediate the expression of inflammatory cytokines, such as IL-6, that flame the tumor microenvironment [10, 11]. In liver cells, IL-6/STAT3 can form a positive feedback loop [12]. In colon cancer, it is a therapeutic target [13]. Similarly, IL-6-activation of the NF-κB pathway has also been shown to contribute to cancer cell growth [14].

Colorectal cancer is the third most common cancer in the world and it is the fourth leading cause of death from all cancer types [15]. During the progress of colorectal cancer, macrophages infiltrate the area of cancerous lesion, are polarized to the M2 phenotype, and subsequently promote the cancer growth [16]. C57BL/6J-Apc^Min/+^ (APC^Min/+^) and mice treated with AOM-DSS are two commonly used mouse models to study the tumorigenesis of colorectal cancer. In APC^Min/+^ mice, intestinal mucosal epithelium can spontaneously develop polyps, which consequently transform to form adenoma and adenocarcinoma. It has been reported that the infiltration of intestinal macrophages in APC^Min/+^ mice is correlated with intestinal tumorigenesis [17]. Analyses of biomarkers such as Arg-1, YM1, Trem2 and CD206 demonstrate the infiltrated macrophages in the intestinal polyps of APC^Min/+^ mice as the M2 phenotype [18]. The AOM-DSS mouse model can be established by intraperitoneal injection with a low dose of carcinogen azoxymethane (AOM) combined with two-cycle treatment of dextran sodium sulphate (DSS).Macrophage infiltration in the intestinal tissue of the AOM-DSS mice was found to be significantly increased during tumorigenesis [19].

Determination of the causal factors in the polarization of macrophages during tumorigenesis would be highly beneficial when developing new forms of cancer therapy [20]. Here, we report that IL-6 secreted from NIH3T3/Src cells plays an important role in the polarization of macrophages. In addition, we show that IL-6 was involved in the development of colorectal cancer in both the APC^Min/+^ and AOM-DSS mouse models. Our findings may offer novel therapeutic strategies for colorectal cancer.

## RESULTS

### Cultured media from NIH-3T3/Src cells and colorectal cancer cells induces polarization of macrophages

**Figure 1:**
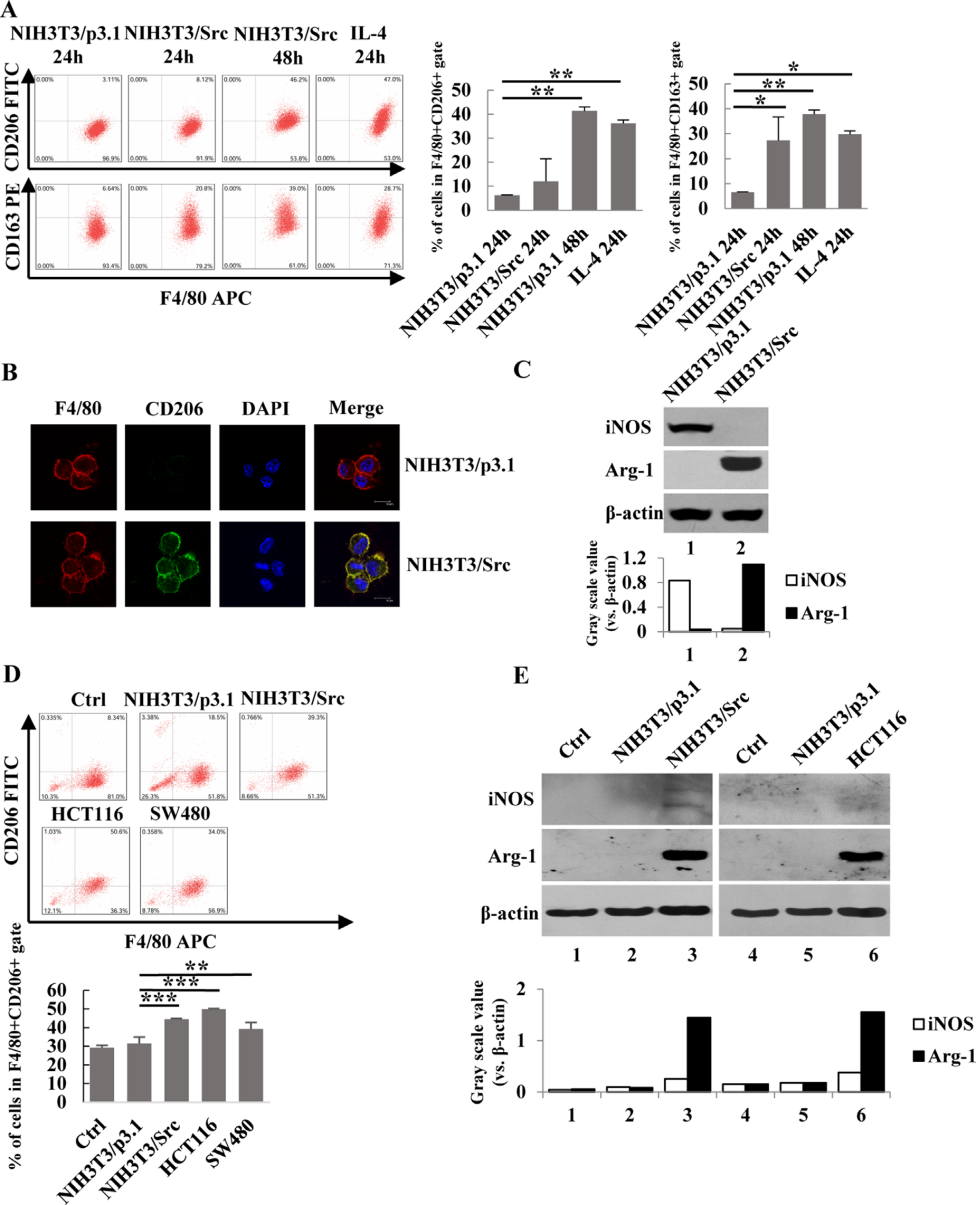
Conditional medium from NIH3T3/Src cells and colon cancer cells induced the polarization of macrophages (**A**) Ana-1 cells were treated with the supernatants from NIH3T3/p3.1, NIH3T3/Src cells or IL-4 (100 ng/ml) for 24 h or 48 h, respectively. The cells were analyzed with anti-F4/80 APC, anti-CD206 FITC and anti-CD163 PE using flow cytometry. Bars represent means ± SD (*n* = 3) for each treatment. ^*^p < 0.05; ^**^p < 0.01; ^***^p < 0.001. (**B**) Ana-1 macrophages were cultured in conditioned medium from NIH3T3/p3.1 or NIH3T3/Src cells for 48 h. Representative immunofluorescence images showed the expression and localization of F4/80 (red) and CD206 (green) in Ana-1 cells. DAPI is shown in blue. (Scale bar: 10 μm). (**C**) Ana-1 cells were cultured in the medium from NIH3T3/p3.1 or NIH3T3/Src for 48 h. The expression of iNOS and Arg-1 protein was analyzed by Western blotting. Intensity was quantified and normalized to β-actin. (**D**) Bone marrow-derived macrophages (BMDM) were cultured in medium from NIH3T3/p3.1, NIH3T3/Src, HCT116 or SW480 cells for 48 h. Cells were stained with anti- F4/80 APC, anti-CD206 FITC, then analyzed using flow cytometry. Bars represent means ± SD (*n* = 3) for each treatment. ^*^p < 0.05; ^**^p < 0.01; ^***^p < 0.001. (**E**) iNOS and Arg-1 expression in BDMD were analyzed by Western blotting. Intensity was quantified and normalized to β-actin.

It is well established that the tumor microenvironment, including infiltrating macrophages, plays a vital role in cancer cell growth. To determine if tumor growth impacts macrophage phenotypes, we constructed a NIH-3T3/Src (Y527F) cell line (NIH-3T3/Src) stably-expressing Src, a proto-oncogene tyrosine-protein kinase and used the conditioned medium of the cells to induce the polarization of Ana-1 cells, a mouse macrophage cell line. In addition, Ana-1 cells treated with IL-4 were used as a positive control. The phenotype of M1 and M2 macrophages was determined by flow cytometry analysis or Western blot analysis using markers for M1 (F4/80/iNOS) and M2 (F4/80/CD206/CD163, Arg-1). As shown in Figure 1A and 1B, the expression of M2 macrophage surface markers CD206 and CD163 was significantly increased as determined by both flow cytometry and immunofluorescence staining. This observation was further confirmed by Western blot analysis to detect the induction of arginase (Arg-1). Arg-1 was dramatically induced in Ana-1 cells cultured with conditioned medium, whereas the expression of iNOS was almost completely suppressed (Figure 1C). To rule out that our observations were specific to cell lines and not primary cells, we isolated mouse bone marrow cells and differentiated them to macrophages (BMDM) with M-CSF. BMDMs were then incubated with either conditioned medium from NIH-3T3/Src cells, human colorectal carcinoma HCT116 cells, or human colorectal adenocarcinoma SW480 cells; polarization of BMDMs was analyzed by flow cytometry for CD206/F4/80. As shown in Figure 1D, the population ratios of CD206/F4/80 (CD11b) labeled cells were 18.5%, 34%, 50.6%, and 39.3% after incubation with NIH-3T3/p3.1, NIH-3T3/Src, HCT116, and SW480 cell culture media, respectively as compared to 8.34% after culture in regular medium. In addition, Arg-1 expression in BMDM was markedly induced by culture with conditioned media from NIH-3T3/p3.1 cells, NIH-3T3/Src cells and HCT116 cells (Figure 1E). Taken together, our results suggest that unknown molecules present in cultured media from cancer cells are able to induce the polarization of M2 macrophages.

### Polarized Ana-1 macrophages promote cancer cell proliferation

**Figure 2:**
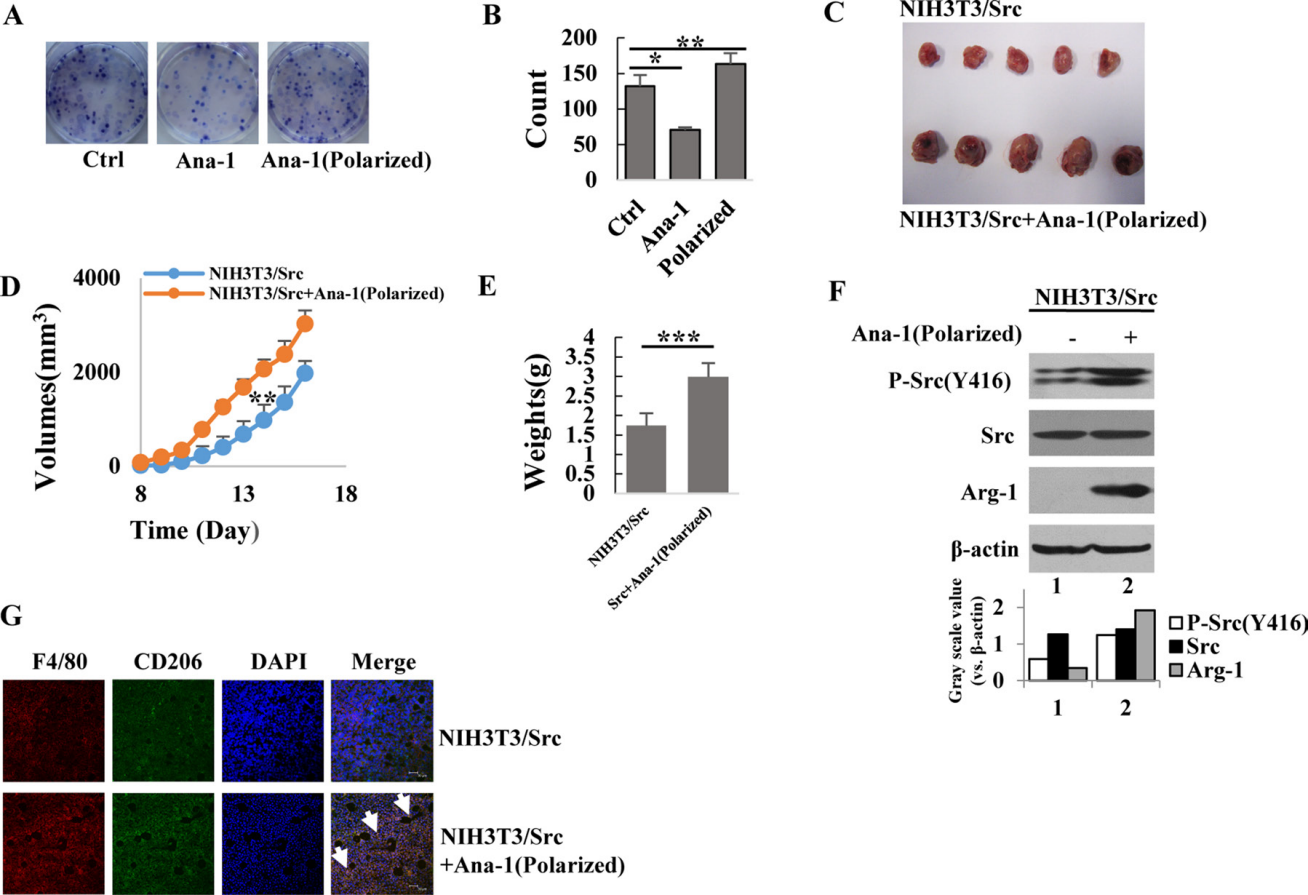
Polarized Ana-1 macrophages promote cancer cell proliferation (**A**–**B**) NIH3T3/Src cells (500) were seeded in six-well plates and treated with supernatants of Ana-1 or polarized Ana-1 macrophages. The colonies were stained two weeks later. Data from representative experiment is shown. Bars represent the colony number of NIH3T3/Src cells. Data are expressed as mean ± SD (*n* = 2), ^*^p < 0.05, ^**^p < 0.01. (**C**) NIH3T3/Src cells (4 × 10^5^) with and without polarized Ana-1 macrophages (8 × 10^4^) were subcutaneously injected into each flank of 4-week old nude mice; mice were sacrificed 16 days later and tumors were shown. (**D**) Xenograft tumor sizes were measured every 2 days with a digital caliper. Data are expressed as mean ± SD (*n* = 5), ^**^p < 0.01. (**E**) Bars represent the weights of xenograft tumors. Data are expressed as mean ± SD (*n* = 5), ^***^p < 0.001. (**F**) The expression of p-Src (Y416), Src and Arg-1 in tumors were analyzed by Western blotting. Intensity was quantified and normalized to β-actin. (**G**) F4/80 and CD206 expression in xenograft tumor tissues. Representative immunofluorescence images showed the expression and localization of F4/80 (red) and CD206 (green). DAPI is shown in blue. The arrows indicated M2 macrophages. (Scale bar: 30 μm).

To determine if the polarized macrophages were able to promote cancer cell proliferation, we grew NIH-3T3/Src cells in the presence of conditioned media from polarized and unpolarized macrophages and quantitated the formation of cell clones by staining the cells with Giemsa. As shown in Figure 2A and 2B, more cell clones developed after culture with conditioned media from polarized M2 macrophages than that in the unpolarized macrophages. Interestingly, the conditional medium from unpolarized macrophages was able to significantly inhibit cancer cell growth; clone numbers were only 50% of that in the control, suggesting that molecules secreted in the culture medium of the unpolarized macrophages suppressed the growth of cancer cells. This observation was further confirmed *in vivo*. NIH-3T3/Src cells mixed with or without polarized M2 Ana-1 macrophages were subcutaneously injected into the flanks of the nude mice. Tumor volumes were measured daily using a caliper (H x W x D) until the animals were euthanized on day 16; then, tumors were dissected and weighed. We found that tumors grew much faster (Figure 2C and 2D) as the average weight of the tumors formed from NIH-3T3/Src cells mixed with M2 macrophages was nearly double the weight of tumors generated from the NIH-3T3/Src cells alone (Figure 2E). Analyses of the tumor tissue extracts showed that the levels of phosphorylated Src and Arg-1 were remarkably higher in tumors derived from NIH-3T3/Src cells mixed with M2 macrophages as compared to NIH-3T3/Src cells alone (Figure 2F). Immunohistostaining for CD206 revealed similar results in tumor tissues (Figure 2G).

### Activation of the NF-κB and JAK/STAT3 pathways is responsible for the high level of IL-6 in the conditional medium of NIH3T3/Src cells

**Figure 3:**
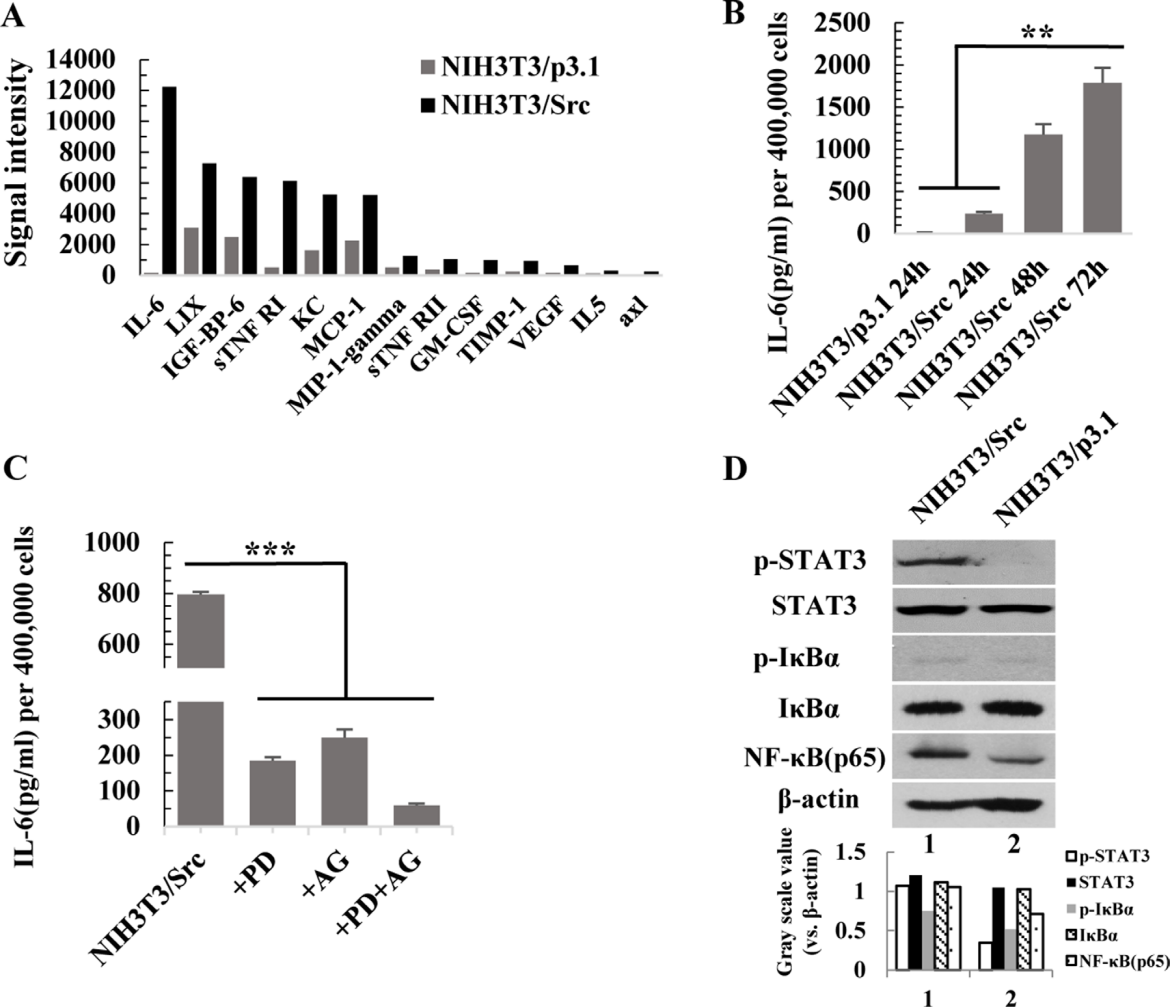
Activation of the NF-κB and JAK/STAT3 pathways is responsible for the high level of IL-6 in the conditional medium from NIH3T3/Src cells (**A**) Cytokines secretion by NIH3T3/p3.1 and NIH3T3/Src cells were measured by AAM-CYT-CYT-3 cytokines antibody array. (**B**) NIH3T3/p3.1 or NIH3T3/Src cells were cultured for 24 h, 48 h or 72 h. The secretion of IL-6 into supernatant was measured by ELISA. Data are expressed as mean ± SD (*n* = 3), ^**^p < 0.01. (**C**) NIH3T3/Src cells were treated with PDTC (100 µM) (PD), AG490 (100 µM) (AG) or PDTC (100 µM) and AG490 (100 µM) (PD/AG) together for 48 h, respectively. Then, the medium was exchanged with fresh medium for another 24 h. The secretion of IL-6 in supernatant was measured by ELISA. Data are expressed as mean ± SD (*n* = 3), ^***^p < 0.001. (**D**) The expression of p-STAT3, STAT3, p-IκBα, IκBα and NF-κB (p65) in NIH3T3/Src and NIH3T3/p3.1 cells were analyzed by Western blotting. Intensity was quantified and normalized to β-actin.

Cancer is a chronic inflammatory disease [21, 22]. To identify secreted cytokines in the conditional medium of NIH-3T3/Src cells, we performed an AAM-CYT-CYT-1 cytokine antibody array. The result was showed in Supplementary Table 1. As shown in Figure 3A, several inflammatory cytokines were present at high levels in the conditional medium of NIH-3T3/Src cells, especially IL-6. The secretion of IL-6 was time dependent (Figure 3B). The NF-κB and JAK/STAT3 pathways mediate inflammatory response in cancer and are associated with poor prognosis in many malignancies [23–26]. To examine if the activation of either pathway was responsible for the induction of IL-6, NIH-3T3/Src cells were grown in the presence or absence of PDTC, a potent chemical inhibitor of the NF-κB pathway, or AG490, a JAK inhibitor, or a combination of both inhibitors, for 24 hr; the level of IL-6 in the medium was measured by ELISA. Our data indicate that the two pathways synergistically contribute to the production of IL-6 (Figure 3C). The remarkably upregulation of NF-κB(p65) was detected in NIH-3T3/Src cells and Src also increased the transcriptional activity of NF-κB(p65) (data not shown). Although the levels of total IκBα and Stat3 were not altered, the obvious up-regulation of p-IκBα and p-Stat3 were detected in NIH-3T3/Src cells compared to NIH-3T3/p3.1 cells (Figure 3D).

### IL-6 induced the polarization of Ana-1 macrophages

**Figure 4:**
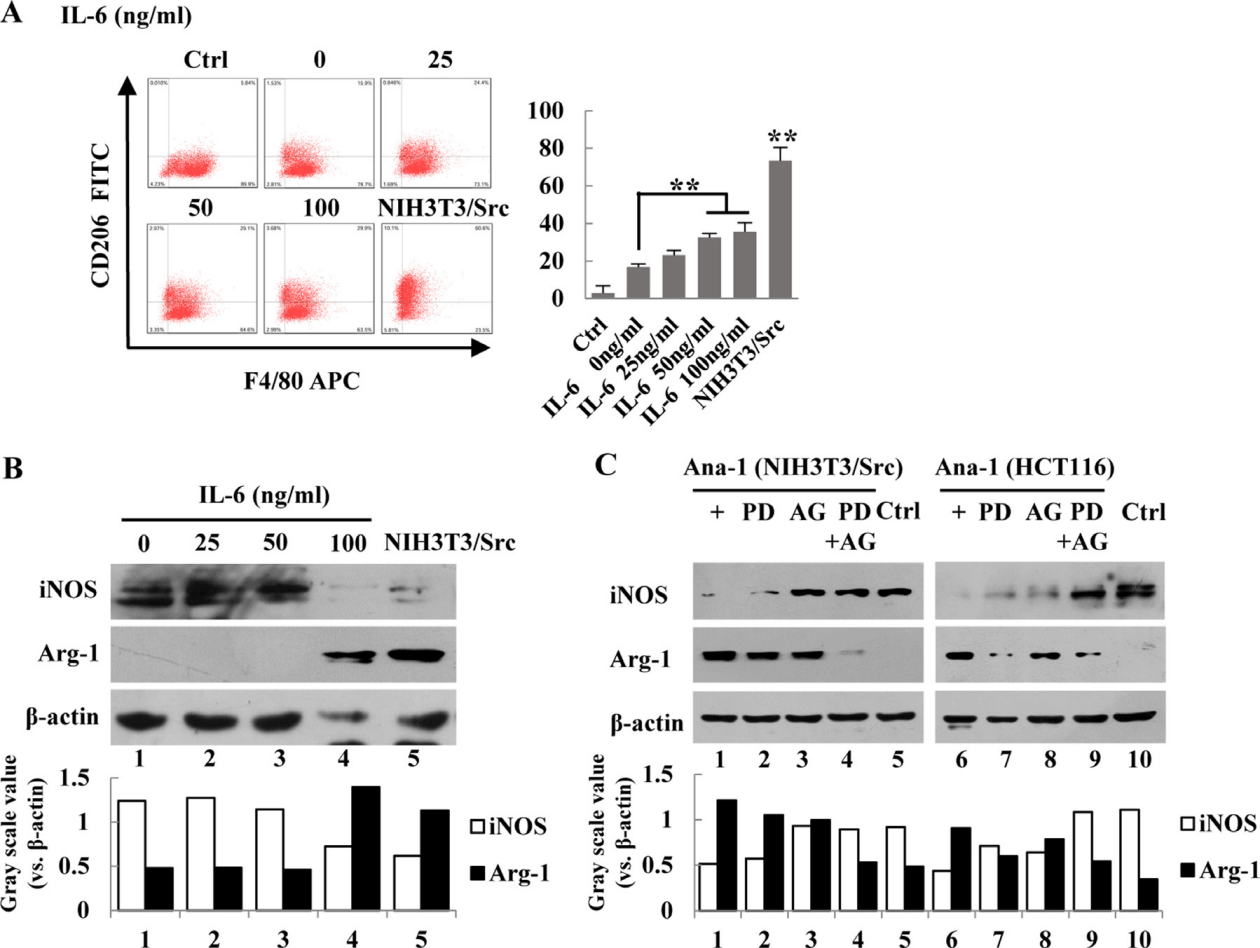
IL-6 induces polarization of Ana-1 macrophages (**A**) Ana-1 cells were treated with different concentration of IL-6 (0, 25, 50, 100 ng/ml), and the supernatants of NIH3T3/Src cells cultured for 48 h. Cells were stained with anti-F4/80 APC and anti-CD206 FITC and analyzed by flow cytometry. Representative data from one experiment is shown. Bars represent means ± SD (*n* = 3) for each treatment. ^*^p < 0.05; ^**^p < 0.01. (**B**) iNOS and Arg-1 expression were analyzed by Western blotting. Intensity was quantified and normalized to β-actin. (**C**) NIH3T3/Src and HCT116 cells were treated with PDTC (100 µM) (PD), AG490 (100 µM) (AG) or PDTC (100 µM) and AG490 (100 µM) (PD+AG) together for 48 h, respectively. Then, the medium was replaced with fresh medium for another 24 h. iNOS and Arg-1 expression were analyzed by Western blotting. Intensity was quantified and normalized to β-actin.

To determine if IL-6 is able to induce the polarization of macrophages, we treated Ana-1 cells with various concentrations of IL-6 for 24 hr; polarization of the cells was determined by flow cytometry for CD206. As shown in Figure 4A, the percentage of CD206-positive cells was increased with higher concentrations of IL-6. Surprisingly, NIH-3T3/Src conditioned media dramatically increased the population of the CD206-positive cells. Next, we examined the expression of Arg-1 (Figure 4B). To determine whether inhibition of the NF-κB and JAK/STAT3 pathways could suppress macrophage polarization, NIH-3T3/Src cells were treated with PDTC or AG490 alone or a combination of both inhibitors and the expression of Arg-1 was determined by Western blot analysis. The inhibitor for each of the pathways alone slightly reduced the levels of Arg-1, but the expression of Arg-1 could be almost completely suppressed after the cells were treated with a combination of the two inhibitors (Figure 4C), suggesting that both pathways are necessary for the induction of IL-6, which subsequently induces macrophage polarization.

### IL-6 contributes to tumorigenesis of colorectal cancer in APCMin/+ and AOM-DSS mice

**Figure 5:**
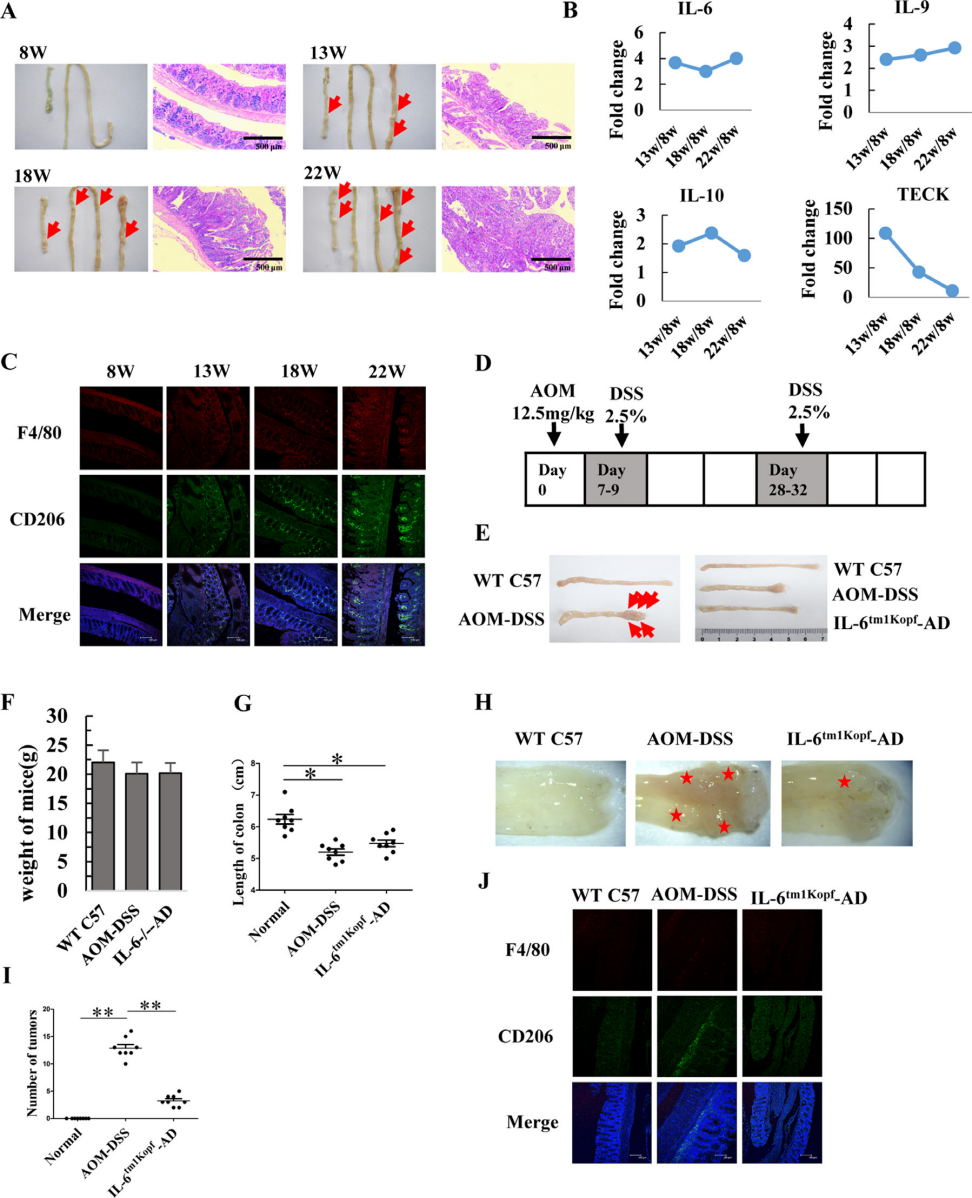
IL-6 contributes to tumorigenesis of colorectal cancer in APCMin/+ and AOM-DSS mice (**A**) Images of normal colon and small intestine as well as pathological sections of colon (100 ×) in 8-, 13-, 18- and 22-week old mice. Arrows indicate the large polyps in colon and small intestinal. (Scale bar: 500 μm). (**B**) Tissue interstitial fluid (TIF) was extracted from colon and measured using an AAM-CYT-G3 cytokines antibody array. Secretion levels of IL-6, IL-9, IL-10, and TECK (thymus expressed chemokine) in the TIF are shown. (**C**) Representative immunofluorescence images indicate the expression and localization of F4/80 (red) and CD206 (green) in colorectal tissues of APC^Min/+^ mice at 8-, 13-, 18- and 22-week of age. DAPI is shown in blue. (Scale bar: 100 μm). (**D**) Schematic diagram showing the AOM-DSS mice model. (**E**) Photos of colon from WT C57 control mice, AOM-DSS treated mice, and IL-6^tm1Kopf^-AOM-DSS treated mice. The arrows indicated colorectal nodules. (**F**) Variation in mouse weight. Data are expressed as mean ± SD (*n* = 8). (**G**) The length of colorectum. Data are expressed as mean ± SD (*n* = 8), ^*^p < 0.05. (**H**) Characterization of colorectum after two DSS cycles. Asterisks indicate the large nodular mass in the colon. (**I**) The number of colorectal nodules. Data are expressed as mean ± SD (*n* = 8), ^**^p < 0.01. (**J**) Representative immunofluorescence images showing the expression and localization of F4/80 (red) and CD206 (green) in colorectal tissues. DAPI is shown in blue. (Scale bar: 100 μm).

To determine the role of IL-6 in the tumorigenesis of colorectal cancer, we established an APC^Min/+^ mice mouse model (Figure 5A) and determined the cytokine profile in tissue interstitial fluid (TIF) by performing an AAM-CYT-G3 cytokine antibody array. In total, 62 cytokines were detected in the TIF (data not shown). As shown in Figure 5B, secreted levels of IL-6, IL-9, and IL-10 remained high in the TIF of 13-, 18-, and 22-week-old mice, which was consistent with an increased population of infiltrating M2 macrophages in the tumors of APC^Min/+^ mice (Figure 5C). To determine whether IL-6 plays an essential role in tumorigenesis of colorectal cancer, we used IL-6^tm1Kopf^ mice, which are IL-6 gene-knockout mice, to generate colorectal neoplasms that result from the inflammatory insult induced by azoxymethane (AOM) and dextran sulfate sodium (DSS) (Figure 5D and 5E). The mice were sacrificed on day 48 after AOM injection, weighed, and intestines were dissected. The body weight of mice in both the AOM-DSS control and the IL-6^tm1Kopf-^AOM-DSS group was slightly decreased (Figure 5F). However, the length of the colon in mice from the AOM-DSS control group (5.2 ± 0.28 cm) was significantly shorter than that in mice from the untreated C57 control group (6.24 ± 0.43 cm). In contrast, the length of the colon in mice from the IL-6^tm1Kopf^-AOM-DSS group (5.48 ± 0.30 cm) was longer than in mice from the AOM-DSS control group (Figure 5E and 5G), implicating that lack of IL-6 may delay tumorigenesis of colorectal cancer. Furthermore, the average number of tumors in the same region of the colon from 8 mice was 13 ± 2 in the AOM-DSS control group and 3 ± 1 in the IL-6^tm1Kopf^-AOM-DSS group (Figure 5H and 5I). Immunohistostaining for CD206 indicated that the population of infiltrating M2 macrophages in sections from colorectal tumors in the AOM-DSS control mice was remarkably higher as well (Figure 5J).

## DISCUSSION

The polarization of macrophages plays an important role in their functions. Upon distinct stimuli, macrophages can be polarized to either the M1 phenotype, which promotes immune responses, or the M2 phenotype which suppresses immune activities [27, 28]. It is well established that macrophages infiltrating into the tumor lesion can be polarized to the M2 phenotype, subsequently promoting tumor growth [1, 2]. However, how macrophages are polarized to the M2 phenotype within the tumor microenvironment remains unclear.

Macrophages can be polarized to the M2 phenotype under certain conditions. Studies have shown that the tumor microenvironment induces the polarization of macrophages toward the M2 phenotype, subsequently promoting tumor growth [29, 30]. It has been reported that the expression of Src in 80% of human colorectal cancer tissues is significantly higher than that of normal colorectal epithelium [31], suggesting that Src plays an important role in tumorigenesis of colorectal tumors [31, 32]. Here, our research interest is to investigate what molecules produced and secreted from colorectal cancer cells, particularly associated with the Src signaling pathway, contribute to macrophage polarization, subsequently promoting cancer growth. Apparently, NIH 3T3 cells over-expressing Src would be better to address the unique role of Src in colorectal cancer growth. Thus, the NIH-3T3/Src cell line is a suitable *in vitro* model of the colorectal cancer microenvironment. Profiling of cytokines and growth factors in the cultured medium of NIH-3T3/Src cells identified IL-6 as a key cytokine for the induction of polarization of macrophages toward the M2 phenotype, although other cytokines and growth factors may also play a role in the event [33, 34].

IL-6 is secreted by a variety of cell types and plays a role in inflammation, response to infection, and wound repair *in vivo* [35, 36]. It has been reported that IL-6 in the microenvironment can induce the expression of the M-CSF receptor on the surface of monocytes [37]. As a result, IL-6 and M-CSF can synergistically promote monocyte differentiation to macrophages [37]. Interestingly, ascites from ovarian cancer patients can also promote monocytes to differentiate into M2 macrophages; further analysis found that the ascites contained a high concentration of IL-6 [38]. In addition, a high level of IL-6 was detected in colorectal carcinoma tissue with liver metastasis [39]. In our study, the distinctive secretion of IL-6 into the cultured media of NIH-3T3/p3.1 and NIH-3T3/Src cells was very significant. It is possible that overexpression of Src in the cells could dramatically stimulate the production of IL-6, suggesting a critical role for the oncogene in manipulating the tumor microenvironment.

Co-expression of NF-κB and STAT3 mediates transcription of inflammatory cytokine genes in tumor cells [40]. High levels of cytokines and chemokines contribute to macrophage polarization in the tumor microenvironment [41, 42]. In our study, the NF-κB and JAK/STAT3 signaling pathways were constitutively activated in NIH3T3/Src cells, leading to a super production of IL-6, which was responsible, at least in part, for induction of macrophage polarization to the M2 phenotype. This event could be inhibited by PDTC and AG490, chemical inhibitors for the NF-κB and JAK/STAT3 pathways, respectively [43–46]. This observation was further confirmed in colorectal cells.

Colorectal cancer is the third most common cancer in the world, and the fourth leading cause of mortality from all malignant tumors [15]. As colorectal cancer progresses, increased macrophage infiltration can be observed in tumor tissue [16]. Analysis of cell surface markers revealed that the majority of infiltrating macrophages in tumor tissue are CD206^+^ cells [47]. In addition, the progress of intestinal tumors in APC^Min/+^ mice was positively associated with the degree of infiltration of macrophages [17]. Macrophages isolated from intestinal tissue of AOM-DSS mice showed a weak anti-inflammatory response and an enhanced effect on promoting tumor growth [20]. Other studies indicate that the level of IL-6 was 10-fold higher in the circulation and was increased by 89% in intestinal polyps in APC^Min/+^ mice at 26 weeks of age [48]. IL-6 was required for development of colitis-associated cancer [49–51] and treatment with anti-IL-6 receptor antibody suppressed polyp growth in APC^Min/+^ mice [52]. All of the evidence strongly suggests that IL-6 is associated with macrophage polarization within the gut microenvironment. In our study, we found that the level of IL-6 in TIF from 8-, 13-, 18- and 22-week APC^Min/+^ mice was correlated with the occurrence and development of colorectal tumors. Obviously, the number of tumors in IL-6^tm1Kopf^mice treated with AOM-DSS was significantly decreased as compared with control mice treated with AOM-DSS. Therefore, suppression of IL-6 may be a potential therapeutic strategy for colorectal tumors.

## MATERIALS AND METHODS

### Cell lines

To construct NIH3T3/Src and NIH3T3/p3.1 cell lines. NIH3T3 cells were transfected with either pcDNA3.1-Src (Y527F) or pcDNA3.1 by using Lipofectamine 2000 transfection reagent (Invitrogen) according to the manufacturer’s instructions. After transformation, cells were grown for 2 weeks with aminoglycoside antibiotic, G418, for selection; and then, drug-resistant colonies were cloned.

NIH3T3, NIH3T3/p3.1, NIH3T3/Src, HCT116, SW480, and Ana-1 cells were cultured in RPMI-1640 (Bioroc™) containing 10% heated-inactivated fetal bovine serum and penicillin-streptomycin. Bone marrow derived macrophage (BMDM) cells were cultured in DMEM (Bioroc™) containing 10% heated-inactivated fetal bovine serum and penicillin-streptomycin. All cells were maintained in a humidified incubator with 5% CO_2_ at 37°C.

Bone marrow cells were isolated from 6 to 8-week old wild-type C57BL/6 mice. Aliquots of 4 × 10^6^ cells per well in six-well plates were cultured in DMEM supplemented with 10% heated-inactivated FBS. The cells differentiated to macrophages in the presence of 10 ng M-CSF.

### Conditioned medium and Macrophages treatment

NIH3T3/p3.1, NIH3T3/Src, colorectal cancer cells (4 × 10^5^) were inoculated in six-well plates. After attachment, the original media were replaced by fresh medium with or without inhibitors for 48 h. NF-κB and JAK/STAT3 signaling was blocked with specific inhibitor ammonium pyrrolidinedithiocarbamate (PDTC) (100 µM; BioVision) and AG490 (100 µM; Calbiochem), respectively. Then, conditioned medium was collected and centrifuged at 3000 rpm for 10 minutes. Ana-1 cells were then incubated with 2 ml conditioned medium for 24 or 48 h. Ana-1 cells treated with IL-4 (100 ng/ml) for 24 or 48 h were used as a positive control.

### Western blotting

Cellular protein was extracted by RIPA Lysis Buffer (Cell Signaling) supplemented with a protease inhibitor cocktail (Applygen). Proteins concentration was determined using the Pierce™ BCA Protein Assay kit (Thermo Scientific). Proteins were subjected to 10% SDS-PAGE and transferred onto nitrocellulose membrane (Schleicher & Schuell). The membranes were blocked with 5% milk and then incubated with antibodies against p-Src (Y416) (2101) (1:1000; Cell Signaling), total-Src (36D10) (1:1000; Cell Signaling), p-STAT3(D3A7) (1:1000; Cell Signaling), total-STAT3 (9132) (1:1000; Cell Signaling), NF-κB(p65) (D14A12) (1:1000; Cell Signaling), Arg-1 (sc-271430) (1:1000; Santa Cruz), or iNOS (sc-650) (1:1000; Santa Cruz), total IκBα (44D4) (1:1000; Cell Signaling), p-IκBα (14D4) (1:1000; Cell Signaling). Antibody incubation was performed overnight at 4°C, membranes were sufficiently washed and then incubated with appropriate HRP-conjugated secondary Antibodys for 1 h at room temperature. Proteins were detected by ECL detection reagent. Expressions of proteins were normalized to β-actin.

### Real-time PCR

Total RNA from cells was obtained using Trizol reagent (Invitrogen) following the manufacturer’s instructions. Extracted RNA was reverse-transcribed to cDNA with PrimeScript™ RT Master Mix (Perfect real time) (Takara). Real-time PCR analysis was performed using Power SYBR Green PCR Master Mix (Applied Biosystems) and detected by the Step-One Plus Real-Time PCR System (Applied Biosystems). All experiments were done in triplicate and relative quantification was done for each sample by normalizing to GAPDH expression. Data were analyzed using Step-One Software. Sequences of primers were listed in Table 1.

**Table 1:** Primers used for quantitative real-time PCR

Identity	Nucleotide sequences	Product size
iNOS	Froward: 5′- GTTCTCAGCCCAACAATACAAGA-3^′^	127bp
	Reverse: 5^′^- GTGGACGGGTCGATGTCAC -3^′^	
IL-4	Reverse: 5^′^- GCCGATGATCTCTCTCAAGTGAT-3^′^	102bp
	Froward: 5^′^-ACTCTGCGCCAGAAACCTC-3^′^	
IL-10	Froward: 5^′^- GCTCTTACTGACTGGCATGAG -3^′^	105bp
	Reverse: 5^′^- CGCAGCTCTAGGAGCATGTG -3^′^	
CD206	Froward: 5^′^-GCTGAATCCCAGAAATTCCGC -3^′^	120bp
	Reverse: 5^′^- ATCACAGGCATACAGGGTGAC-3^′^	
Arg-1	Froward: 5^′^- TGGCTTGCGAGACGTAGAC-3^′^	160bp
	Reverse: 5^′^- GCTCAGGTGAATCGGCCTTTT-3^′^	
IL-12	Froward: 5^′^-ACTCTGCGCCAGAAACCTC-3^′^	111bp
	Reverse: 5^′^- CACCCTGTTGATGGTCACGAC-3^′^	
GAPDH(h)	Froward:5^′^-GCACCGTCAAGGCTGAGAAC-3^′^	130bp
	Reverse: 5^′^-TGGTGAAGAACGCCAGTGGA-3^′^	
GAPDH(m)	Froward: 5^′^- AGGTCGGTGTGAACGGATTTG -3^′^	123bp
	Reverse: 5^′^- TGTAGACCATGTAGTTGAGGTCA -3^′^	

### Flow cytometry assays

Bone marrow-derived macrophages (BMDM) and Ana-1 cells were cultured in six-well plates, treated with conditioned media from NIH3T3/p3.1, NIH3T3/Src or colorectal cancer cells as described previously. Cells were harvested and washed 3 times with PBS, then resuspended in 100 µl 0.5% PBS and stained using monoclonal antibodies: APC-conjugated anti-mouse F4/80 (BioLegend), FITC-conjugated anti-mouse CD206 (BioLegend) and PE-conjugated anti-mouse CD163 (eBioscience) for 30 min in an ice bath. Isotype control antibodies (BioLegend and eBioscience) were used as negative controls. Flow cytometric analysis was performed on LSRII (BD). Data was analyzed with FlowJo software (TreeStar).

### Cytokine secreting and ELISA

Secretion of cytokines from the supernatants of cells was measured with an AAM-CYT-CYT-3 RayBio Mouse Cytokine Antibody Array. Cytokines in the tissue interstitial fluid (TIF) of APC^Min/+^ mice were also measured with an AAM-CYT-CYT-G3 RayBio Mouse Cytokine Antibody Array. Cytokine levels in supernatants were assayed using an appropriate mouse ELISA kit (R&D systems). A standard curve was plotted with increasing concentrations of cytokines. Supernatants were incubated in a 96-well microliter plate, and the levels of cytokines were assayed.

### Immunofluorescence

Paraffin sections were deparaffinized in a graded xylene series (15 min each), and hydrated in a graded ethanol series (3 min each). Sections were washed with PBS and treated with 3% H_2_O_2_ solution in PBS at room temperature for 10 min to quench endogenous peroxidase activity. Frozen sections were fixed in pre-cooled acetone (-20^o^C) for 10 min. Ana-1 cells were placed on glass slides and fixed in 4% paraformaldehyde for 15 min. Then, sections were respectively blocked by incubation with 5% bovine serum albumin in PBS, and incubated with mouse anti-F4/80 (red) (1:500 dilution; Abcam), anti-CD206 (green) (1:500 dilution; Abcam) and DAPI (blue) (1:500 dilution; Applygen Technologies). Secondary antibodies conjugated to Dylight 488 or 649 were used (1:500 dilution; EarthOx Life Sciences). Fluorescent images were obtained using Laser Scanning Confocal Microscope.

### Animal experiments

APC^Min/+^ male mice and WT C57BL/6J female mice were purchased from Jackson Laboratory. IL-6^tm1Kopf^ mice were purchased from Model Animal Research Center of Nanjing University at 6–8 weeks of age. The establishment of the AOM-DSS mouse model was conducted as previously described [51]. Briefly, azoxymethane (AOM) (12.5 mg/kg; Sigma-Aldrich) was injected intraperitoneally into 18–20g weight mice. After 7 days, 2.5% Dextran sodium sulfate (DSS) (NW36, 000–50,000Da, MP Biomedicals) in drinking water feeding mice for 5 days followed by normal drinking for 16 days. This DSS treatment was repeated for one additional cycle. Two DSS cycles was required to construct AOM-DSS mice model.

Cells of implanted tumors were harvested by treatment with 0.05% trypsin–EDTA and then resuspended in PBS. Balb/c athymic nude female mice (4-week old) were injected with 150 µl of the cell suspension subcutaneously, which contained 1 × 10^6^ 3T3/Src cells with or without 2 × 10^5^ macrophages per mouse. Tumor sizes were measured every 2 days. Mice were sacrificed and tumor weights were measured 16 days after injection.

All animals handled in strict accordance with good animal practice as defined by Beijing Municipal Science and Technology Commission; all animal work was approved by this committee.

### Preparation of tissue interstitial fluid (TIF)

APC^Min/+^ mice were sacrificed and the colon tissue of each mouse was opened longitudinally and washed with PBS to push out any remaining colon contents. The colon tissue was cut into small pieces (< 3 mm^3^) with a pair of scissors and washed three times with PBS (8 ml each time). After last wash, the pieces were placed into a new tube with 200 µl PBS and 5 µl of a proteinase inhibitor cocktail (Calbiochem/Merck Millipore); tubes were incubated for 1 h at 37°C with constant mixing. Step-wise centrifugation (1,000 g for 5 minutes, 5,000 g for 10 min and 12,000 g for 10 min at 4°C) was performed to remove cells or debris; the supernatant containing proteins was carefully transferred to a new tube each step. Subsequent experiments occurred immediately or the samples were immediately store at –80°C for future use.

### Statistical analysis

All data were presented as the means ± SD. All experiments were repeated at least two or three times; data from one representative experiment is depicted. Results between treatment and control groups were compared using two-tailed Student’s *t-*tests. A *P*-value < 0.05 was considered to be statistically significant.

## SUPPLEMENTARY MATERIALS TABLE





## References

[1] Chanmee T, Ontong P, Konno K, Itano N (2014). Tumor-associated macrophages as major players in the tumor microenvironment. Cancers.

[2] Alderton GK (2010). Tumour microenvironment: Macrophages lead the way. Nat Rev Cancer.

[3] Mantovani A, Bottazzi B, Colotta F, Sozzani S, Ruco L (1992). The origin and function of tumor-associated macrophages. Immunol Today.

[4] Lewis CE, Pollard JW (2006). Distinct role of macrophages in different tumor microenvironments. Cancer Res.

[5] Gordon S, Martinez FO (2010). Alternative activation of macrophages: mechanism and functions. Immunity.

[6] Sica A, Mantovani A (2012). Macrophage plasticity and polarization: *in vivo* veritas. J Clin Invest.

[7] Karin M, Lin A (2002). NF-κB at the crossroads of life and death. Nat Immunol.

[8] Richmond A (2002). NF-κB, chemokine gene transcription and tumour growth. Nat Rev Immunol.

[9] Wang T, Niu G, Kortylewski M, Burdelya L, Shain K, Zhang S, Bhattacharya R, Gabrilovich D, Heller R, Coppola D, Dalton W, Jove R, Pardoll D, Yu H (2004). Regulation of the innate and adaptive immune responses by Stat-3 signaling in tumor cells. Nat Med.

[10] Balkwill F (2003). Chemokine biolog y in cancer. Seminars in immunology.

[11] Nishimoto N, Kishimoto T (2006). Interleukin 6: from bench to bedside. Nat Clin Pract Rheumatol.

[12] He G, Dhar D, Nakagawa H, Font-Burgada J, Ogata H, Jiang Y, Shalapour S, Seki E, Yost SE, Jepsen K, Frazer KA, Harismendy O, Hatziapostolou M (2013). Identification of liver cancer progenitors whose malignant progression depends on autocrine IL-6 signaling. Cell.

[13] Wang SW, Sun YM (2014). The IL-6/JAK/STAT3 pathway: potential therapeutic strategies in treating colorectal cancer (Review). Int J Oncol.

[14] Iliopoulos D, Hirsch HA, Struhl K (2009). An epigenetic switch involving NF-kappaB, Lin28, Let-7 MicroRNA, and IL6 links inflammation to cell transformation. Cell.

[15] Wiseman M (2008). The second World Cancer Research Fund/American Institute for Cancer Research expert report. Food, nutrition, physical activity, and the prevention of cancer: a global perspective. Proc Nutr Soc.

[16] Bailey C, Negus R, Morris A, Ziprin P, Goldin R, Allavena P, Peck D, Darzi A (2007). Chemokine expression is associated with the accumulation of tumour associated macrophages (TAMs) and progression in human colorectal cancer. Clin Exp Metastasis.

[17] Kettunen HL, Kettunen AS, Rautonen NE (2003). Intestinal immune responses in wild-type and Apcmin/+ mouse, a model for colon cancer. Cancer Res.

[18] Nakanishi Y, Nakatsuji M, Seno H, Ishizu S, Akitake-Kawano R, Kanda K, Ueo T, Komekado H, Kawada M, Minami M (2011). COX-2 inhibition alters the phenotype of tumor-associated macrophages from M2 to M1 in ApcMin/+ mouse polyps. Carcinogenesis.

[19] Lai CS, Tsai ML, Cheng AC, Li S, Lo CY, Wang Y, Xiao H, Ho CT, Wang YJ, Pan MH (2011). Chemoprevention of colonic tumorigenesis by dietary hydroxylated polymethoxyflavones in azoxymethane-treated mice. Mol Nutr Food Res.

[20] Wang W, Li X, Zheng D, Zhang D, Peng X, Zhang X, Ai F, Wang X, Ma J, Xiong W, Li G, Zhou Y, Shen S (2015). Dynamic changes and functions of macrophages and M1/M2 subpopulations during ulcerative colitis-associated carcinogenesis in an AOM/DSS mouse model. Mol Med Rep.

[21] Coussens LM, Werb Z (2002). Inflammation and cancer. Nature.

[22] Colotta F, Allavena P, Sica A, Garlanda C, Mantovani A (2009). Cancer-related inflammation, the seventh hallmark of cancer: links to genetic instability. Carcinogenesis.

[23] Kim JY, Jung HH, Ahn S, Bae S, Lee SK, Kim SW, Lee JE, Nam SJ, Ahn JS, Im YH, Park YH (2016). The relationship between nuclear factor (NF)-κB family gene expression and prognosis in triple-negative breast cancer (TNBC) patients receiving adjuvant doxorubicin treatment. Sci Rep.

[24] Annunziata CM, Stavnes HT, Kleinberg L, Berner A, Hernandez LF, Birrer MJ, Steinberg SM, Davidson B, Kohn EC (2010). Nuclear factor κB transcription factors are coexpressed and convey a poor outcome in ovarian cancer. Cancer.

[25] Kim DY, Cha ST, Ahn DH, Kang HY, Kwon CI, Ko KH, Hwang SG, Park PW, Rim KS, Hong SP (2009). STAT3 expression in gastric cancer indicates a poor prognosis. J Gastroenterol Hepatol.

[26] Thomas S, Snowden J, Zeidler M, Danson S (2015). The role of JAK/STAT signalling in the pathogenesis, prognosis and treatment of solid tumours. Br J Cancer.

[27] Ruffell B, Affara NI, Coussens LM (2012). Differential macrophage programming in the tumor microenvironment. Trends Immunol.

[28] Martinez FO, Gordon S (2014). The M1 and M2 paradigm of macrophage activation: time for reassessment. F1000Prime Rep.

[29] DeNardo DG, Barreto JB, Andreu P, Vasquez L, Tawfik D, Kolhatkar N, Coussens LM (2009). CD4+ T cells regulate pulmonary metastasis of mammary carcinomas by enhancing protumor properties of macrophages. Cancer Cell.

[30] Zaynagetdinov R, Sherrill TP, Polosukhin VV, Han W, Ausborn JA, McLoed AG, McMahon FB, Gleaves LA, Degryse AL, Stathopoulos GT, Yull FE, Blackwell TS (2011). A critical role for macrophages in promotion of urethane-induced lung carci nogenesis. J Immunol.

[31] Chen J, Elfiky A, Han M, Chen C, Saif MW (2014). The role of Src in colon cancer and its therapeutic implications. Clin Colorectal Cancer.

[32] Hirai H, Varmus HE (1990). SH2 mutants of c-src that are host dependent for transformation are trans-dominant inhibitors of mouse cell transformation by activated c-src. Genes Dev.

[33] Nio Y, Yamauchi T, Iwabu M, Okada-Iwabu M, Funata M, Yamaguchi M, Ueki K, Kadowaki T (2012). Monocyte chemoattractant protein-1 (MCP-1) deficiency enhances alternatively activated M2 macrophages and ameliorates insulin resistance and fatty liver in lipoatrophic diabetic A-ZIP transgenic mice. Diabetologia.

[34] Selleri S, Bifsha P, Civini S, Pacelli C, Dieng MM, Lemieux W, Jin P, Bazin R, Patey N, Marincola FM, Moldovan F, Zaouter C, Trudeau LE (2016). Human mesenchymal stromal cell-secreted lactate induces M2-macrophage differentiation by metabolic reprogramming. Oncotarget.

[35] Hunter CA, Jones SA (2015). IL-6 as a keystone cytokine in health and disease. Nat Immunol.

[36] Tanaka T, Narazaki M, Kishimoto T (2014). IL-6 in inflammation, immunity, and disease. Cold Spring Harb Perspect Biol.

[37] Chomarat P, Banchereau J, Davoust J, Palucka AK (2000). IL-6 switches the differentiation of monocytes from dendritic cells to macrophages. Nat Immunol.

[38] Duluc D, Delneste Y, Tan F, Moles MP, Grimaud L, Lenoir J, Preisser L, Anegon I, Catala L, Ifrah N, Descamps P, Gamelin E, Gascan H (2007). Tumor-associated leukemia inhibitory factor and IL-6 skew monocyte differentiation into tumor-associated macrophage-like cells. Blood.

[39] Li YY, Hsieh LL, Tang RP, Liao SK, Yeh KY (2009). Interleukin-6 (IL-6) released by macrophages induces IL-6 secretion in the human colon cancer HT-29 cell line. Hum Immunol.

[40] Narayan C, Kumar A (2012). Constitutive over expression of IL-1beta, IL-6, NF-kappaB, and Stat3 is a potential cause of lung tumorgenesis in urethane (ethyl carbamate) induced Balb/c mice. J Carcinog.

[41] Lu H, Ouyang W, Huang C (2006). Inflammation, a key event in cancer development. Mol Cancer Res.

[42] Daley JM, Reichner JS, Mahoney EJ, Manfield L, Henry WL, Mastrofrancesco B, Albina JE (2005). Modulation of macrophage phenotype by soluble product(s) released from neutrophils. J Immunol.

[43] Ziegler-Heitbrock H, Sternsdorf T, Liese J, Belohradsky B, Weber C, Wedel A, Schreck R, Bäuerle P, Ströbel M (1993). Pyrrolidine dithiocarbamate inhibits NF-kappa B mobilization and TNF production in human monocytes. J Immunol.

[44] Liu SF, Ye X, Malik AB (1999). Inhibition of NF-κB activation by pyrrolidine dithiocarbamate prevents *in vivo* expression of proinflammatory genes. Circulation.

[45] Teng Y, Ghoshal P, Ngoka L, Mei Y, Cowell JK (2013). Critical role of the WASF3 gene in JAK2/STAT3 regulation of cancer cell motility. Carcinogenesis.

[46] Burdelya L, Catlett-Falcone R, Levitzki A, Cheng F, Mora LB, Sotomayor E, Coppola D, Sun J, Sebti S, Dalton WS, Jove R, Yu H (2002). Combination therapy with AG-490 and interleukin 12 achieves greater antitumor effects than either agent alone. Mol Cancer Ther.

[47] Kemp R, Norton S, Taylor E, Dunn E, Munro F, Black M, McCall J (2015). Colorectal tumour associated macrophages are more pro-inflammatory than adjacent control bowel tissue macrophage populations (TUM6P. 1003). J Immunol.

[48] Baltgalvis KA, Berger FG, Pena MM, Davis JM, Muga SJ, Carson JA (2008). Interleukin-6 and cachexia in ApcMin/+ mice. Am J Physiol Regul Integr Comp Physiol.

[49] Grivennikov S, Karin E, Terzic J, Mucida D, Yu GY, Vallabhapurapu S, Scheller J, Rose-John S, Cheroutre H, Eckmann L (2009). IL-6 and Stat3 are required for survival of intestinal epithelial cells and development of colitis-associated cancer. Cancer Cell.

[50] White JP, Baynes JW, Welle SL, Kostek MC, Matesic LE, Sato S, Carson JA (2011). The regulation of skeletal muscle protein turnover during the progression of cancer cachexia in the Apc min/+ mouse. PLoS One.

[51] Greten FR, Eckmann L, Greten TF, Park JM, Li ZW, Egan LJ, Kagnoff MF, Karin M (2004). IKKβ links inflammation and tumorigenesis in a mouse model of colitis-associated cancer. Cell.

[52] Yaoita T, Sasaki Y, Yokozawa J, Sato T, Kanno N, Sakuta K, Yagi M, Yoshizawa K, Iwano D, Nagino K, Nomura E, Abe Y, Nishise S (2015). Treatment with anti-interleukin-6 receptor antibody ameliorates intestinal polyposis in Apc(Min/+) mice under high-fat diet conditions. Tohoku J Exp Med.

