# Supplementary material for: IL-6 influences the polarization of macrophages and the formation and growth of colorectal tumor
Source: Oncotarget. 2018 Apr 3;9(25):17443–54. doi: 10.18632/oncotarget.24734 (PMC5915127; doi:10.18632/oncotarget.24734)
Supplement: Supplementary file 1 [file oncotarget-09-17443-s001.pdf]

# IL-6 influences the polarization of macrophages and the formation and growth of colorectal tumor

## SUPPLEMENTARY MATERIALS

**Supplementary Table 1: Cytokine profiles of NIH3T3/p3.1 and NIH3T3/Src cells.** See_ Supplementary_Table 1.
