# Supplementary material for: IL-6 influences the polarization of macrophages and the formation and growth of colorectal tumor
Source: Oncotarget. 2018 Apr 3;9(25):17443–54. doi: 10.18632/oncotarget.24734 (PMC5915127; doi:10.18632/oncotarget.24734)
Supplement: Supplementary file 2 [file oncotarget-09-17443-s002.docx]

**Table S1 Cytokine profiles of NIH3T3/p3.1 and NIH3T3/Src cells**

| Cytokine | Supernatant  of  NIH3T3/p3.1cells | Supernatant  of  NIH3T3/Src cells |
| --- | --- | --- |
| Axl (Anexelekto) | 77.72 | 244.30 |
| BLC (B-lymphocyte chemoattractant) | 770.64 | 868.80 |
| CD30L | 110.47 | 108.80 |
| CD30 | 556.97 | 662.30 |
| CD40 | 88.10 | 89.30 |
| CRG-2 (cytokine responsive gene-2) | 176.76 | 188.30 |
| CTACK (Cutaneous T-cell attracting chemokine) | 799.79 | 814.30 |
| CXCL16 (C-X-C motif chemokine ligand 16) | 300.97 | 340.30 |
| Eotaxin | 137.63 | 128.80 |
| Eotaxin-2 | 858.10 | 773.80 |
| Fas ligand | 301.77 | 294.30 |
| Fractalkine | 228.28 | 232.80 |
| G-CSF (Granulocyte-colony stimulating factor) | 286.19 | 337.30 |
| GM-CSF (Granulocyte-macrophage colony-stimulating factor) | 165.58 | 983.80 |
| IFN-gamma (interferon-gamma) | 197.53 | 239.80 |
| IGF-BP-3 (insulin like growth factor binding protein-3) | 451.93 | 569.80 |
| IGF-BP-5 | 167.98 | 173.30 |
| IGF-BP-6 | 2,496.34 | 6,372.30 |
| IL1-alpha (interleukin1- alpha） | 721.51 | 694.30 |
| IL1-beta | 190.74 | 126.30 |
| IL2 | 140.02 | 141.30 |
| IL3 | 82.11 | 101.80 |
| IL3 R beta | 235.47 | 301.30 |
| IL4 | 537.00 | 716.80 |
| IL5 | 137.23 | 315.30 |
| IL6 | 172.37 | 12,253.80 |
| IL9 | 390.43 | 403.80 |
| IL10 | 193.54 | 202.80 |
| IL12-p40/p70 | 137.23 | 153.80 |
| IL12-p70 | 805.78 | 691.80 |
| IL13 | 180.76 | 183.80 |
| IL17 | 46.57 | 72.30 |
| KC (Keratinocyte chemoattractant) | 1,627.30 | 5,264.30 |
| Leptin R | 380.45 | 437.80 |
| Leptin | 274.21 | 336.80 |
| LIX (lipopolysaccharide-induced CXC chemokine) | 3,108.98 | 7,258.30 |
| L-Selectin | 235.87 | 324.80 |
| Lymphotactin | 714.72 | 972.30 |
| MCP-1 (Monocyte Chemoattractant Protein-1) | 2,253.12 | 5,233.30 |
| MCP-5 | 446.74 | 501.30 |
| M-CSF (macrophage colony-stimulating factor) | 732.30 | 881.30 |
| MIG (Monokine induced by interferon gamma) | 340.91 | 330.80 |
| MIP-1-alpha (macrophage inflammatory protein1-alpha) | 120.45 | 118.30 |
| MIP-1-gamma | 510.64 | 1,278.30 |
| MIP-2 | 714.72 | 847.80 |
| MIP-3-beta | 323.73 | 319.80 |
| MIP-3-alpha | 197.13 | 263.30 |
| PF4 (Platelet factor 4) | 777.03 | 924.30 |
| P-Selectin | 863.69 | 1,064.80 |
| RANTES (Regulated on Activation, Normal T Cell Expressed and Secreted) | 358.88 | 383.30 |
| SCF (Stem Cell Factor) | 259.83 | 247.80 |
| SDF-1-alpha (stromal cell-derived factor 1) | 1,017.05 | 1,208.30 |
| TARC (Thymus- and activation-regulated chemokine) | 187.95 | 217.30 |
| TCA-3 (tricarboxylic acid) | 711.53 | 697.30 |
| TECK (thymocyte expressed chemokine) | 121.65 | 124.30 |
| TIMP-1 (Tissue Inhibitor of  Metalloproteinases- l) | 259.04 | 926.30 |
| TNF-alpha (tumor necrosis factor alpha) | 248.25 | 280.80 |
| sTNF RI (Human soluble tumor necrosis factor receptor I) | 519.83 | 6,132.80 |
| sTNF RII (Human soluble tumor necrosis factor receptor II) | 395.62 | 1,049.30 |
| TPO (Thrombopoietin) | 389.63 | 390.80 |
| VCAM-1 (vascular cell adhesion molecule 1) | 185.95 | 184.80 |
| VEGF (Vascular endothelial growth factor) | 173.57 | 672.80 |
